# Identification of von Willebrand Factor-Enriched Small Extracellular Vesicles as a Blood-Based Biomarker for the Detection of Head and Neck Squamous Cell Carcinoma

**DOI:** 10.3390/cancers18142339

**Published:** 2026-07-20

**Authors:** Yue Su, Kekoolani S. Visan, Sunyoung Ham, Xuanxuan Li, Su-Ho Park, Cherrie W. K. Ng, Judy Wai Ping Yam, Jason Y. K. Chan, Andreas Möller

**Affiliations:** 1 Department of Otorhinolaryngology, Head and Neck Surgery, Faculty of Medicine, The Chinese University of Hong Kong, Shatin, Hong Kong SAR, China; yuesu@link.cuhk.edu.hk (Y.S.); sunyoungham@cuhk.edu.hk (S.H.); xuanxuanli@link.cuhk.edu.hk (X.L.); su-ho.park@cuhk.edu.hk (S.-H.P.); cherrieng@cuhk.edu.hk (C.W.K.N.); jasonchan@ent.cuhk.edu.hk (J.Y.K.C.); 2 JC STEM Lab of Personalised Cancer Medicine, Li Ka Shing Institute of Health Sciences, The Chinese University of Hong Kong, Hong Kong SAR, China; 3 Department of Pathology, School of Clinical Medicine, Li Ka Shing Faculty of Medicine, The University of Hong Kong, Hong Kong SAR, China; judyyam@pathology.hku.hk; 4 State Key Laboratory of Liver Research (The University of Hong Kong), Hong Kong SAR, China; 5 Materials Innovation Institute for Life Sciences and Energy (MILES), The University of Hong Kong Shenzhen Institute of Research and Innovation (HKU-SIRI), Shenzhen 518045, China

**Keywords:** small extracellular vesicles, head and neck cancer, head and neck squamous cell carcinoma, von Willebrand factor, diagnosis, biomarker

## Abstract

**Background**: Head and neck squamous cell carcinoma (HNSCC) remains a major global health challenge due to the lack of effective and non-invasive diagnostic tools, often resulting in late-stage detection of cancer. Small extracellular vesicles (sEVs) have emerged as promising biomarkers for early cancer detection and disease monitoring due to their omnipresence and stability in bodily fluids, such as blood plasma. In addition, cancer-derived sEVs specifically carry cargo reflective of oncogene-derived molecular alterations. In summary, these characteristics position sEVs as a potential platform for non-invasive testing of HNSCC. **Methods**: Plasma-derived sEVs from HNSCC patients (*n* = 71) and benign subjects (*n* = 25) were isolated using size exclusion chromatography. Proteomic profiling via liquid chromatography-tandem mass spectrometry identified potential candidate biomarkers, followed by ELISA validation. **Results**: Proteomic analyses revealed a significant enrichment of multiple proteins in HNSCC-derived sEVs compared to sEVs derived from non-cancer individuals. The von Willebrand factor (vWF) was significantly higher in HNSCC patient-derived sEVs compared to those derived from benign individuals. A validation cohort confirmed that sEV-associated vWF (sEV-vWF) effectively distinguished HNSCC patients from benign subjects, demonstrating strong diagnostic performance, specifically in laryngeal HNSCC (AUC = 0.82) and oropharyngeal HNSCC (AUC = 0.96) patient cohorts. Moreover, postoperative reductions and recurrence-associated increases in sEV-vWF levels corresponded with clinical outcomes, indicating its potential as a dynamic disease indicator. **Conclusions**: These findings highlight sEV-vWF as a novel and non-invasive biomarker with potential applications in early detection and real-time monitoring of HNSCC, supporting advancement toward precision liquid biopsy strategies in head and neck oncology.

## Simple Summary

Head and neck squamous cell carcinoma (HNSCC) is often diagnosed late because current tests are invasive and not suitable for routine screening. In this study, we investigated whether small extracellular vesicles (sEVs), nano-sized particles released into the plasma, serve as a source of non-invasive biomarkers for HNSCC. We found that the von Willebrand factor (vWF) was significantly enriched in sEVs from HNSCC patients, especially those with laryngeal and/or oropharyngeal tumours, but not in whole plasma. SEV-associated vWF also decreased after surgery and increased again in some patients with recurrence. These findings suggest that sEV-vWF could support non-invasive detection and monitor HNSCC using a simple blood test.

## 1. Background

Head and neck cancer (HNC) represents a complex group of malignancies that arise from the epithelial tissues of the oral cavity, pharynx, and larynx [1]. HNC is the seventh most common cancer worldwide, with high prevalence amongst Eastern and Southern Asian populations [2]. The most common HNC subtype is head and neck squamous cell carcinoma (HNSCC) [3]. According to the Global Cancer Observatory, in 2022, there were approximately 890,000 new cases and 450,000 HNSCC-related deaths, with incidence rates continuing to increase [4,5].

Current diagnostic methods for HNSCC mainly rely on visual inspection, gene-based tests, imaging (computed tomography (CT) or magnetic resonance imaging (MRI)), invasive biopsies [6], and human papillomavirus (HPV)-based plasma ctDNA analysis [7]. Despite the multiple diagnostic approaches available, there is no consensus regarding routine screening protocols for HNSCC. Current diagnostic methods are invasive, prone to high sampling errors, and do not provide real-time assessments, resulting in delays in diagnosis and treatment initiation. HNSCC patients generally have a poor prognosis, with an overall five-year survival rate of 35–70% [8]. Approximately 50–70% of patients are diagnosed at late stages (stage III or IV) and at an average age of 50–70 years [9,10]. However, the incidence of HNSCC, especially oral cavity squamous cell carcinoma (OCSCC) and oropharyngeal squamous cell carcinoma (OPSCC), is steadily increasing in young individuals under the age of 45 [11]. Notably, up to 50% of HNSCC patients with advanced disease experience metastasis and recurrence, resulting in a high mortality rate [12]. Although recent therapies have yielded modest improvements in survival rates for late-stage HNSCC, overall prognosis remains poor, and advancements in long-term survival rates remain minimal [13]. This indicates an urgent demand for innovative, non-invasive diagnostic tools that can facilitate earlier detection rates and enhance the accuracy of HNSCC diagnoses.

Small extracellular vesicles (sEVs) have emerged as promising candidates as cancer biomarkers [14,15]. The sEVs are membrane-enclosed lipid bilayer vesicles of endocytic origin and are less than 200 nm in diameter. They are released by all cells, including cancer cells, containing a cargo rich in lipids, nucleic acids, and proteins largely specific to their parental cells [16]. Recent research has revealed that sEVs mediate bidirectional communication between cancer cells and the tumour microenvironment, resulting in tumour dissemination and progression [17,18]. Blood sEVs are primarily derived from platelets and other blood vessel cells but also include sEVs derived from various tissues, including cancer masses [19,20,21].

Plasma-derived sEVs offer distinct advantages for biomarker development due to their easy accessibility and potential to provide insight into tumour biology in real time. The sEV lipid bilayer membrane protects internal content from environmental degradation; hence, they are more stable than free proteins circulating in plasma [22]. These proteins affect key biological processes, such as cell growth and immune response [23]. Since tumour cells continuously release sEVs into the circulation, sEV proteins can reflect the dynamic changes occurring in the cancer, thereby enabling real-time monitoring of disease, making them accessible for convenient, efficient, and non-invasive collection [24]. Furthermore, while sEVs have been reported in HNSCC, the correlation among specific protein signatures identified in sEVs, early-stage HNSCC, and clinical application remains incompletely defined [25,26,27,28]. Biomarkers allow for the monitoring of disease progression and response to therapy, enabling timely adjustments in treatment plans. However, there are still limited studies on the use of EV protein biomarkers in HNSCC.

Here, we report the proteomic evaluation of plasma-derived sEV HNSCC samples, identifying an enrichment of the von Willebrand factor (vWF). The vWF is a large multimeric haemostatic glycoprotein found in plasma. We show that plasma-contained sEV-vWF could distinguish OPSCC patients from benign individuals with a sensitivity and specificity of 100% and 88%, respectively, as well as laryngeal squamous cell carcinoma (LSCC) patients from benign individuals with a sensitivity and specificity of 73.68% and 76%, respectively. Additionally, our work demonstrated the potential of sEV-vWF to detect cancer recurrence in HNSCC patients. This may enable not only accurate diagnoses but also personalized therapies in individuals with a propensity for metastasis or recurrence.

## 2. Materials and Methods

### 2.1. Patient Characteristics

A total of 96 participants were included in this study, including 25 individuals with benign head and neck disease and 71 HNSCC patients. The HNSCC patient cohort consisted of both early-stage (stages I and II, *n* = 24) and late-stage (stages III and IV, *n* = 47) patients, enabling the assessment of candidate biomarkers for early cancer detection. The characteristics of the participants are detailed in Table 1. The majority of the HNSCC patients were males (59/71, 84.3%), similar to the gender distribution amongst individuals with benign head and neck disease. The age distribution was predominantly over 50 years old, with a median age of 64 (range 41–86), with a significant difference in the mean age between the HNSCC patients and benign individuals.

Among the HNSCC patients were former or current smokers (47/71, 69.1%) and former or current drinkers (44/71, 62%). The primary sites of cancer in the cohort were the oral cavity (24/71, 33.8%), hypopharynx (12/71, 16.9%), larynx (28/71, 39.4%), and oropharynx (7/71, 9.9%).

As patient sample collection was performed from 2015 to 2023, tumour staging was determined according to the AJCC staging system in use at the time of diagnosis (AJCC staging 7th edition for cases diagnosed prior to 2018; AJCC staging 8th edition for cases diagnosed in 2018 and thereafter).

### 2.2. Blood Collection and Plasma Isolation

Whole blood was collected by the Department of Otorhinolaryngology, Head and Neck Surgery of the Prince of Wales Hospital, Hong Kong, in accordance with the protocol approved by The Joint Chinese University of Hong Kong–Hospital Authority New Territories East Cluster Clinical Research Ethics Committee (2015.396). All blood donors provided consent. Plasma was isolated from 25 benign subjects and 71 HNSCC patients, as previously described [29]. In brief, the whole blood was centrifuged; after 30 min, it was placed at room temperature at 1200× *g* at 4 °C; after 10 min in a Beckman Coulter Allegra V-15R Benchtop Centrifuge, the plasma supernatant was transferred to a new tube. Centrifugation for 10 min at 1800× *g* at 4 °C was used to deplete platelet contamination in the plasma sample. The resulting plasma was snap-frozen and stored at −80 °C.

### 2.3. sEV Isolation and Analysis

The isolation of human plasma-derived sEVs was performed using size exclusion chromatography (SP1-B-100, qEVoriginal, 70 nm, Izon Science Ltd., Christchurch, New Zealand), as previously reported [30,31]. Briefly, plasma was centrifuged at 10,000× *g* 4 °C for 10 min. Then, 500 µL of plasma was overlaid on top of the size exclusion column, followed by elution with 400 µL fractions of phosphate-buffered saline (PBS) (14190250, ThermoFisher, Hong Kong, China). The sEV-containing fractions (F7–F12) were concentrated by Amicon^®^ Ultra-4 10 kDa centrifugal filter units (UFC801096, Merck Millipore, Darmstadt, Germany) via centrifugation at 4000× *g* at 4 °C in a Beckman Coulter Allegra V-15R Benchtop Centrifuge. The final sEV volume was stored at −80 °C.

### 2.4. Particle Quantification Analysis

The size and quantification of sEVs were evaluated using ZetaView (Particle Metrix, Meerbusch, Germany) software ZetaView (version 8.05.16 SP7) according to the manufacturer’s instructions (equipped with 488 nm laser and 80% sensitivity) [30]. Concentrations of approximately 10^7^ particles/mL were injected into the cell assembly. Three independent analyses were performed for each sample, and the average size and concentration of particles were calculated.

### 2.5. Transmission Electron Microscopy

Purified sEV morphology was assessed, as previously described [31]. Briefly, samples were fixed in 2% paraformaldehyde, then loaded on a 200-mesh carbon-coated copper grid (01801, Ted Pella, Redding, CA, USA), and incubated for 5 min, negatively stained with 10 µL 4% uranyl acetate (22400, Electron Microscopy Sciences, Morgantown, PA, USA) for 2 min, washed with filtered distilled water, and the grid was dried before imaging with a Hitachi (Hong Kong, China) HT7700 Transmission Electron Microscope (acc. voltage = 100 kV, emission = 10.2 µA).

### 2.6. Western Blot Analysis

Cells were lysed with radio immunoprecipitation assay lysis and extraction buffer (89901, ThermoFisher) with 10% protease inhibitor (11697498001, Merck Millipore). Protein concentrations of cell lysate and sEV preparations were quantified by Bradford assay (5000205, Bio-Rad, Hercules, CA, USA). A total of 5 × 10^9^ sEV particles were loaded onto 10% SDS gel and transferred to an immunoblot PVDF membrane (1620177, Bio-Rad Corporation) using the Trans-Blot Turbo System (1704150, Bio-Rad Corporation), as previously described [30]. Mouse anti-CD81 (1:1000, sc166029, Santa Cruz, Dallas, TX, USA), rabbit anti-CD9 (1:1000, 13174S, Cell Signaling Technology, Danvers, MA, USA), rabbit anti-GAPDH (1:1000, 2118S, Cell Signaling Technology), and rabbit anti-Calnexin (1:1000, 2679S, Cell Signaling Technology, Danvers, MA, USA) antibodies were used for immunoblotting. Subsequently, incubation with the secondary antibodies, anti-mouse HRP (1:30,000, 7076S, Cell Signaling Technology, Danvers, MA, USA) and anti-rabbit HRP (1:10,000, 7074S, Cell Signaling Technology, Danvers, MA, USA), was performed. Proteins were visualised using the JESS chemiluminescent blot imaging system (Bio-Techne, Minneapolis, MN, USA).

### 2.7. Enzyme-Linked Immunosorbent Assay (ELISA)

To assess biomarker performance using ELISA, an initial pilot test using 5 benign and 5 HNSCC patients was conducted. For validation of biomarker performance, analysis was expanded to a larger cohort, including those used in the initial pilot test.

The abundance of vWF in human plasma and plasma-derived sEVs was measured by human vWF ELISA kit (EHVWF, ThermoFisher), human Thrombospondin-1 ELISA kit (DY3074, R&D Systems, Minneapolis, MN, USA), human Galectin-3BP/MAC-2BP ELISA kit (DY2226, R&D Systems), Human Nidogen-1 ELISA kit (DY2570, R&D Systems), human LBP ELISA kit (DY870-05, R&D Systems), human Syndecan-1 ELISA kit (ab47352, Abcam, Cambridge, UK), human Desmoglein-1 ELISA Kit (EH153RB, ThermoFisher), human Hemicentin-1 (HMCN1) ELISA Kit (RK11097, ABclonal, Woburn, MA, USA), and human IRE1 ELISA kit (HUFI02015, Assay Genie, Dublin, Ireland) according to the manufacturer’s protocol.

### 2.8. Proteomics

The proteomic profiling of plasma-derived sEVs was performed, as previously described [32]. Briefly, sEVs (20 µg) derived from the plasma of benign (*n* = 5) and HNSCC (*n* = 5) individuals were lysed and denatured in EasyPep lysis buffer (Thermo Fisher Scientific) (10 min at 95 °C) and then digested with LysC/trypsin (180 min at 37 °C). Peptides were separated by the Dionex Ultimate3000 nanoRSLC system and coupled to a Thermo Fisher Orbitrap Fusion Tribid Lumos mass spectrometer (ThermoFisher Scientific, Hong Kong, China). Peptides were separated on a commercial C18 column (75 µm i.d × length 50 cm × particle size 2 µm), which was used in series with a NanoTrap column (75 µm i.d × length 2 cm × particle size 3 µm) (Thermo Fisher Scientific). Trypsin specificity was set to two missed cleavages and with a false discovery rate of 1% for both proteins and PSM. Raw mass spectrometry data were processed using Spectronaut. Direct DIA data analysis was performed on Spectronaut v.14 using default settings without N-acetyl variable modification enabled [33]. Significant differences in sEV protein abundance between HNSCC patients and benign individuals were determined by setting a fold change (FC) threshold ≥ 2 and a significance value of *p* < 0.05.

### 2.9. Statistical Analysis

Mann–Whitney tests (non-parametric) and one-way analysis of variance (ANOVA) were utilized for statistical analyses. The diagnostic efficiency of vWF was assessed using the area under the curve (AUC) of the receiver operating characteristic curve. All experiments were conducted with three biological repeats and comprised at least three technical replicates. Error bars represent mean ± standard error of mean (SEM), and *p* < 0.05 was considered statistically significant (* *p* < 0.05; ** *p* < 0.01; *** *p* < 0.001).

## 3. Results

### 3.1. Proteomic Evaluation of Plasma sEVs Identified Significant Differences Between HNSCC Patients and Benign Individuals

**Figure 1:**
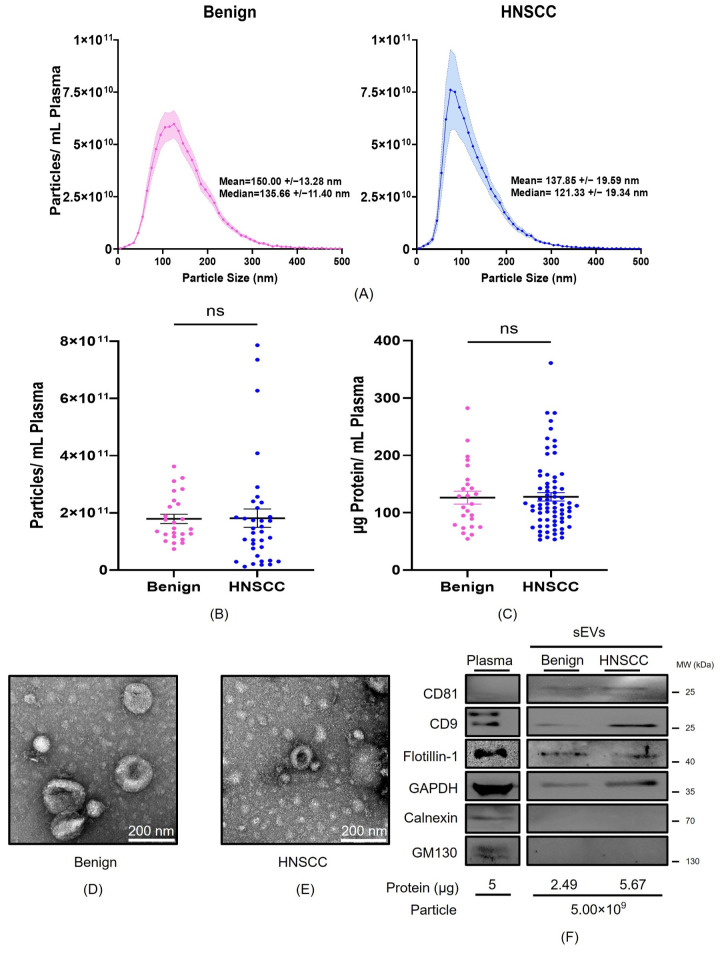
Characterization of sEVs isolated from the plasma of benign individuals and HNSCC. Nanoparticle tracking analysis determined the (**A**) size distribution and (**B**) particle concentration of EVs in each group. (**C**) Protein quantification indicated crude protein concentration of EVs in each group. Representative transmission electron microscopy images of EVs are isolated from (**D**) benign head and neck disease, and (**E**) HNSCC patients’ plasma shows a lipid bilayer and cup-shaped structure. Scale bar = 200 nm. (**F**) Representative Western blots showing detection or absence of CD81, CD9, Flotillin-1, Calnexin, and GM130 for EVs separated from plasma, compared with whole plasma. Data are presented as the mean ± SEM, ns, not significant. Statistical analyses were performed using unpaired *t*-test. HNSCC: head and neck squamous cell carcinoma. The original Western blot figures can be found in Supplementary File S1.

Plasma-derived sEVs were enriched by size exclusion chromatography from benign individuals (*n* = 25) and patients with HNSCC (*n* = 71). The sEVs isolated were quantified and analysed by nanoparticle tracking analysis (NTA) and Bradford assay, respectively, revealing no significant differences in particle and protein concentrations, as well as size distribution (Figure 1A–C). Visualisation by transmission electron microscopy demonstrated that the sEVs isolated from both benign individuals and HNSCC patients have a spherical-like structure, with a diameter smaller than 200 nm, consistent with the size distribution measured by NTA (Figure 1D,E). Western blot analysis detected sEV protein markers Flotillin-1, CD81, and CD9. However, the cell lysate markers, Calnexin (endoplasmic reticulum marker) and GM130 (Golgi marker), were not detected in either benign or HNSCC-derived sEVs (Figure 1F). Taken together, the morphological and biochemical results validated the successful isolation of intact sEVs from plasma of both benign individuals and HNSCC patients.

**Figure 2:**
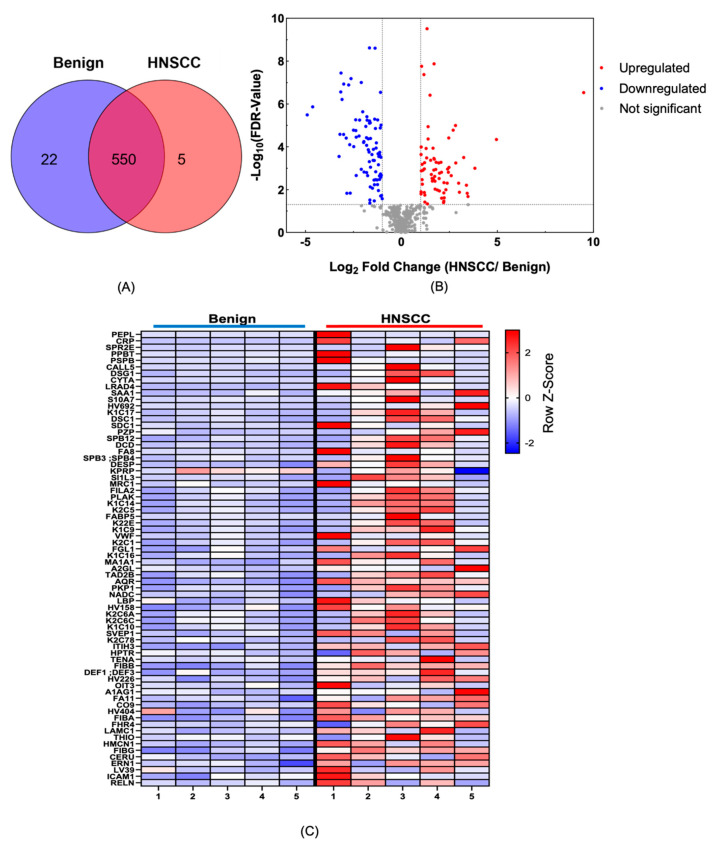
Proteomic profiling of plasma-derived sEVs of benign individuals and HNSCC, using DIA mass spectrometry. (**A**) Venn diagram of a total of 577 proteins detected in patients with benign head and neck disease and HNSCC. (**B**) Volcano plot of quantitative mass spectrometry identifying 70 proteins that are increased in HNSCC patient plasma-derived sEVs (FDR < 0.005; *n* = 5). (**C**) Heatmap demonstrating quantitative mass spectrometry identification of 70 proteins significantly increased in sEVs derived from HNSCC patient plasma. The colour key denotes the row z-score. HNSCC: head and neck squamous cell carcinoma. Note: the full list of proteins detected is provided in Supplementary Table S1.

To understand the proteomic differences of plasma sEVs between benign and HNSCC patients and discover potential biomarkers, liquid chromatography-mass spectrometry (LC-MS/MS) was carried out using five representative plasma sEV samples from each group. Proteomic analysis identified 577 proteins, of which 22 were uniquely found in sEVs from benign individuals, whilst five were specific only to HNSCC patient sEVs. The remaining 550 proteins were detected in both groups (Figure 2A and Figure S1, Table S1), with 70 proteins significantly enriched and 86 proteins significantly reduced in HNSCC patient sEVs compared to sEVs from benign individuals (Figure 2B,C).

### 3.2. vWF in sEVs Can Detect HNSCC and Recurrence After Therapeutic Intervention

To identify potential HNSCC biomarkers, we selected six proteins that were elevated in HNSCC sEVs, as detected by mass spectrometry, including Lipopolysaccharide binding protein (LBP), Desmoglein-1 (DSG1), Hemicentin-1 (HMCN1), Syndecan-1 (SDC1), Inositol requiring enzyme 1 (IRE1), and vWF. We also evaluated the diagnostic capacity of three proteins, previously identified by our group as prognostic biomarkers for lung malignancies, including Thrombospondin-1 (THBS1), Mac-2 binding protein (MAC-2BP), and Nidogen-1 (NID1) [34,35]. These nine proteins were tested by ELISA in a small independent cohort consisting of 21 benign individuals and 21 HNSCC patients.

**Figure 3:**
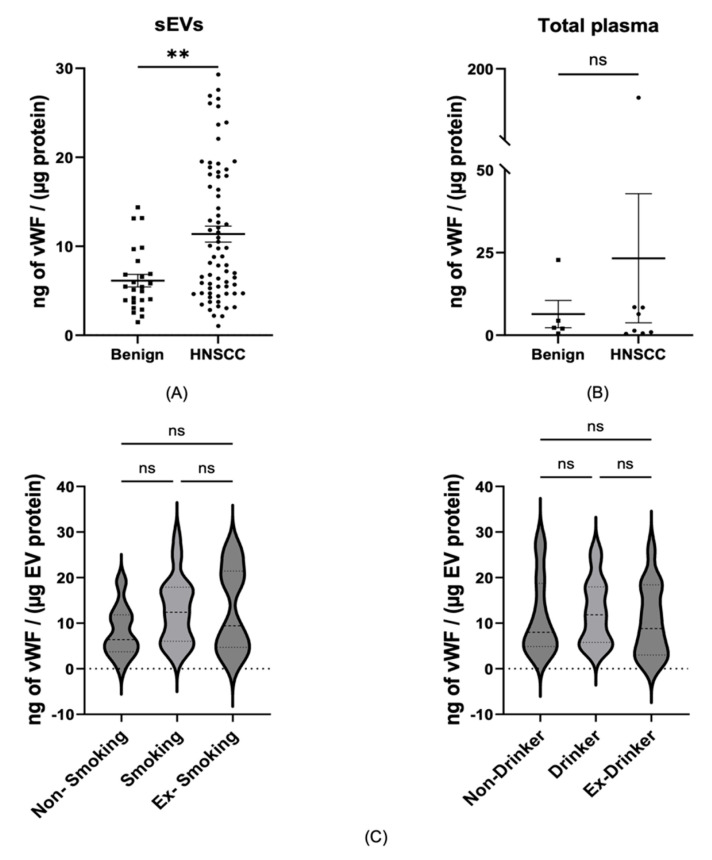
ELISA-based validation confirms increased sEV-vWF in HNSCC patients compared to benign individuals. vWF concentration in (**A**) plasma-derived sEVs (HNSCC patients (*n* = 71); benign individuals (*n* = 25)) and (**B**) plasma (HNSCC patients (*n* = 8); benign individuals (*n* = 5)) as measured by ELISA. Violin plot displaying sEV-vWF levels according to (**C**) smoking status (non-smoking, smoking, ex-smoking) and alcohol consumption status (non-drinker, drinker, ex-drinker). HNSCC: head and neck squamous cell carcinoma. Data were shown as mean ± SEM, ** *p* < 0.01, ns, not significant. Statistical analyses were performed using unpaired *t*-test.

All potential protein biomarkers, except for vWF, were ineffective in distinguishing benign individuals from HNSCC patients. Both DSG1 and IRE1 were undetectable, whereas THBS1, MAC-2BP, NID1, LBP, SDC1, and HMCN1 showed no significant difference in abundance between sEVs derived from benign individuals and HNSCC patients (Figure S2). Overall, NID1, LBP, and HMCN1 were very low in abundance, which may have masked subtle disease-related changes (Figure S2C,E,F). Intriguingly, vWF was significantly increased in HNSCC patient sEVs compared to sEVs derived from benign subjects (Figure 3A).

The vWF is a predominantly soluble plasma glycoprotein and circulates in the blood as a multimeric complex [36]. To determine whether this difference in vWF was specific to proteins co-isolated with sEVs, whole plasma was assessed for potential differences in abundance between benign individuals and HNSCC patients. ELISA revealed no significant difference in concentrations of plasma vWF between benign individuals and HNSCC patients (Figure 3B), suggesting that the elevated vWF detected in sEVs reflects a specific enrichment associated with sEVs rather than differences in circulating plasma levels.

To evaluate the efficacy of sEV-vWF as a potential HNSCC biomarker, a larger validation cohort consisting of 25 benign individuals and 71 HNSCC patients was assessed (Figure 3A). Smoking and alcohol consumption are major risk factors for HNSCC, which may independently affect plasma proteomic panels [37]. However, there was no correlation between sEV-vWF abundance and smoking or drinking status, which demonstrates sEV-vWF as an independent marker for HNSCC status (Figure 3C).

To assess the potential confounding effect of age, patients were stratified by age, and sEV-vWF levels were analysed (Figure S3). Because the benign and HNSCC cohorts differed in age distribution, we additionally examined an age-restricted subgroup (40–60 years) to improve comparability between groups. Elevated sEV-vWF levels in HNSCC patients remained statistically significant in the < 60 years and 40–60 years subgroups (Figure S3A). In patients aged ≥ 60 years, sEV-vWF levels also showed an increasing trend in HNSCC compared with benign, although this did not reach statistical significance (*p* = 0.0897), likely due to the limited number of benign samples in this age group (*n* = 4) (Figure S3A).

We further examined the association between age and sEV-vWF abundance. No significant correlation was observed in the benign group (r = −0.1244, *p* = 0.5534). The HNSCC cohort displayed a clear age-dependent upward trend but showed a modest yet significant correlation between age and vWF abundance (r = 0.2530, *p* = 0.0333). Although the correlation coefficient is modest (r = 0.3415), the association between age and vWF levels was statistically significant (*p* = 0.0007) (Figure S3B). Stratification by tumour subtype showed that OPSCC exhibited the highest sEV-vWF levels in the age < 60 years group, and LSCC exhibited the highest sEV-vWF levels in the age ≥ 60 years group (Figure S3C).

**Figure 4:**
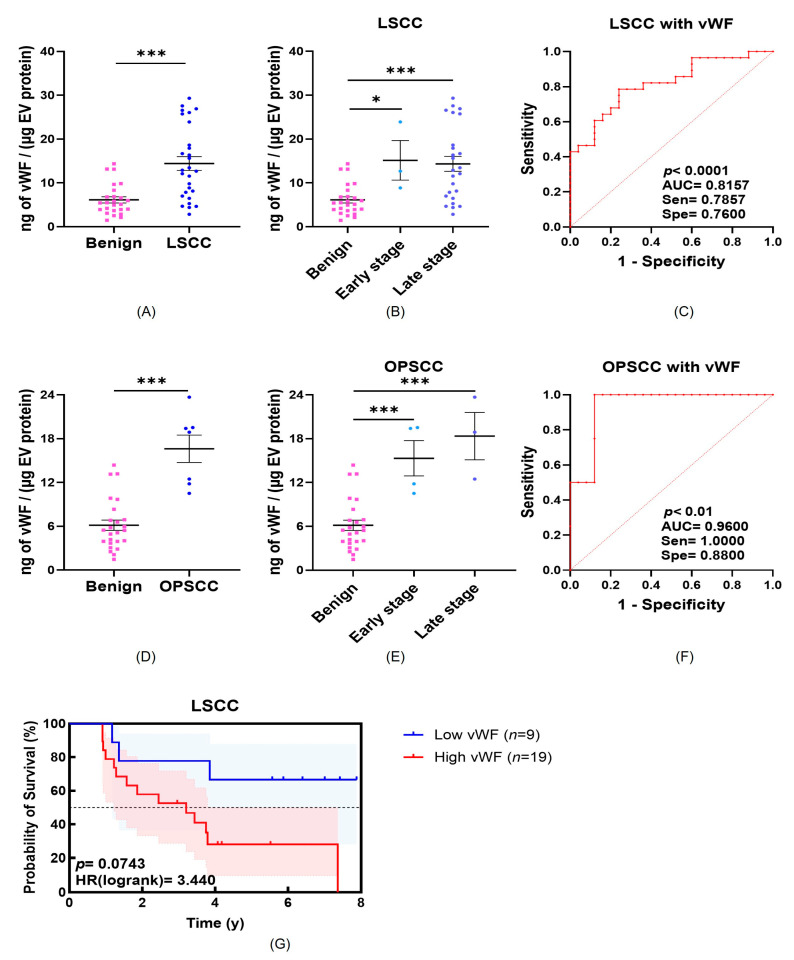
Plasma-derived sEV-vWF distinguished early- and late-stage LSCC and OPSCC patients from benign individuals. Abundance of sEV-vWF in (**A**) LSCC patients (*n* = 28) compared to benign individuals (*n* = 25); (**B**) early- (*n* = 3) and late-stage (*n* = 25) LSCC patients compared to benign individuals (*n* = 25). (**C**) ROC curves of sEV-vWF abundance in LSCC patients. Abundance of sEV-vWF in (**D**) OPSCC patients (*n* = 7) compared to benign individuals (*n* = 25); (**E**) early- (*n* = 4) and late-stage (*n* = 3) OPSCC patients compared to benign individuals (*n* = 25). (**F**) ROC curves of sEV-vWF expression levels in OPSCC patients. (**G**) Probability of overall survival of LSCC patients based on sEV-vWF abundance (low (*n* = 9) vs. high (*n* = 19)). Data were shown as mean ± SEM, * *p* < 0.05, *** *p* < 0.001. Statistical analyses were performed using unpaired *t*-test. LSCC: laryngeal squamous cell carcinoma; OPSCC: oropharyngeal squamous cell carcinoma.

In addition to the diagnostic capacity of sEV-vWF for HNSCC, we evaluated the potential of vWF to distinguish specific HNSCC subtypes. HNSCC patients were categorised by subtypes, including LSCC, OPSCC, OCSCC, and hypopharyngeal squamous cell carcinoma (HSCC). Interestingly, ELISA revealed that vWF abundance was significantly increased in LSCC-derived sEVs (*n* = 28) compared to benign individuals (*n* = 25; Figure 4A). Of the 28 LSCC patients, three had early-stage and 25 had late-stage cancers. The abundance of vWF was significantly increased in both early-stage and late-stage LSCC sEVs (Figure 4B). ROC curve analysis illustrated an AUC of 0.8157, with a 95% confidence interval (CI) of 0.7025 to 0.9289, allowing for significant discrimination of LSCC from benign individuals (*p* < 0.0001), with a sensitivity of 78.6% and specificity of 76% (Figure 4C).

Similarly, there was a significant enrichment of sEV-vWF in OPSCC patients (*n* = 7) compared to benign individuals (Figure 4D). In the OPSCC cohort, four patients had early-stage and three patients had late-stage cancers. The abundance of vWF was significantly increased in both early- and late-stage OPSCC sEVs (Figure 4E). However, given the small number of patients in each stage group, these findings should be considered exploratory. The performance of sEV-vWF had an AUC of 0.96 (*p* < 0.01, 95% CI, 0.8975 to 1.000), with a sensitivity of 100% and specificity of 88% (Figure 4F), in differentiating OPSCC from benign individuals; given the limited sample size, these estimates should be interpreted with caution. However, the abundance of sEV-vWF in HSCC patients (Figure S4A) and OCSCC patients did not significantly differ from benign individuals (Figure S4B). In summary, sEV-vWF could potentially serve as an effective diagnostic marker for LSCC and OPSCC, even at their early stages. Next, we investigated the capacity of sEV-vWF abundance to monitor disease recurrence. First, to evaluate the association between sEV-vWF abundance and patient survival rates, Kaplan–Meier curves were employed. Elevated plasma sEV-vWF levels showed a trend towards poorer overall survival of LSCC patients (*p* = 0.0743, Hazard Ratio (log rank) = 3.440, 95% CI = 1.164, 9.199; Figure 4G), although not statistically significant.

HPV status is a critical driver in determining HNSCC subtype and prognostic outcomes, particularly OPSCC [38]. However, data regarding HPV status was not available for all patients included in the analyses, with only 18 patients having undergone HPV testing, with four p16-positive and 14 p16-negative cases. The abundance of sEV-vWF was similar between HPV-negative and HPV-positive HNSCC patients (Figure S4C). Amongst the OPSCC subgroup, HPV data was available for only one patient, precluding appropriate statistical analysis, and interpretation of findings according to HPV status.

**Figure 5:**
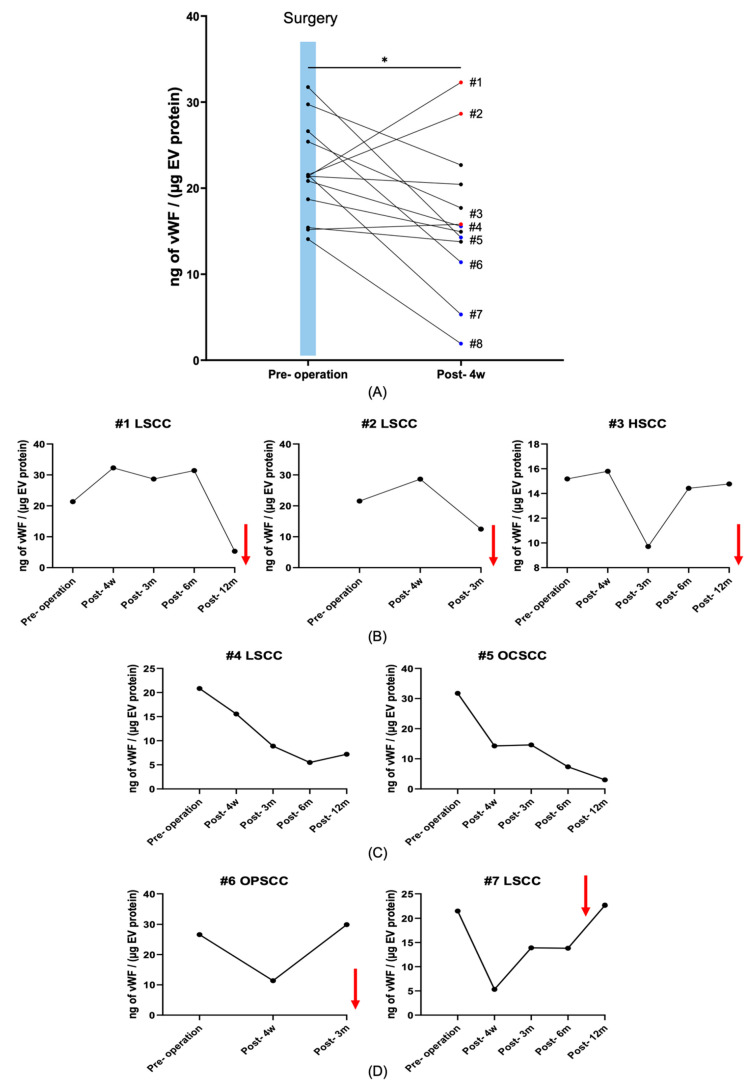
The abundance of sEV-vWF in the blood of individuals with HNSCC correlates with recurrence. (**A**) Plasma-derived sEV-vWF concentrations in 13 HNSCC patients (each dot represents an individual patient) before and after surgical resection of primary tumours. Assessment of sEV-vWF abundance at baseline (pre-surgery, black dot) and 4 weeks (red/blue dot), 3 months, 6 months, and 12 months post-surgical resection in (**B**) three patients whose sEV-vWF abundance increased after surgery, #1 LSCC and #3 HSCC patients experienced recurrence after one year, and #2 LSCC patient experienced recurrence within 6 months after surgery. (**C**) Two patients who did not experience recurrence within one year post-surgery. (**D**) Two patients whose sEV-vWF abundance decreased to 4 weeks after surgery and experienced recurrence within one year post-surgery. Data were shown as mean ± SEM. * *p* < 0.05. Statistical analyses were performed using paired *t*-test, Wilcoxon test. LSCC: laryngeal squamous cell carcinoma, HSCC: hypopharyngeal squamous cell carcinoma; OCSCC: oral cavity squamous cell carcinoma; OPSCC: oropharyngeal squamous cell carcinoma. The red dot represents the sEV-vWF abundance increased after surgery; the blue dot represents the sEV-vWF abundance decreased dramatically after surgery. The red arrow points to the time of recurrence.

To evaluate the potential of sEV-vWF in monitoring disease recurrence, we assessed its abundance across multiple clinical time points in patients. Blood was collected from 13 HNSCC patients at baseline (pre-surgery) and 4 weeks post-surgical resection. Measurements of sEV-vWF at pre-surgery and 4 weeks post-surgery by ELISA demonstrated a significant decrease in vWF in 10 of the 13 HNSCC patients (Figure 5A). However, another three patients (#1 and #2 LSCC patients, and #3 HSCC patient) exhibited an increasing trend of sEV-vWF abundance after surgery to 4 weeks post-surgery. Interestingly, these patients showed a fluctuating trend of sEV-vWF abundance post-surgery and experienced disease recurrence after surgery (Figure 5B). Longitudinal analysis further suggested the association between persistently low sEV-vWF levels after surgery. Patient #4 (LSCC) and patient #5 (OCSCC), who remained free of recurrence during the follow-up period, showed a sustained low sEV-vWF level over time, with levels decreasing from pre-surgery to 6 months post-surgery (Figure 5C). In contrast, #6 OPSCC and #7 LSCC patients, who experienced recurrence within 12 months post-surgery, exhibited an increasing trend of sEV-vWF abundance from 4 weeks post-surgery and despite the #7 LSCC patient having undergone radiotherapy 2 months post-surgery (Figure 5D). This trend suggests that longer-term lower sEV-vWF abundance may be associated with a favourable prognosis and that plasma sEV-vWF could be used as an early indicator of recurrence in HNSCC patients (Figure 5D). The sEV-vWF abundance in the #8 LSCC patient dropped markedly after surgery and showed no signs of recurrence during the available follow-up period; however, this patient lacked long-term blood follow-up, and only pre- and post-surgery two-timepoint samples were available (Figure 5A). Collectively, these data underscore the potential of sEV-vWF as both a diagnostic and disease-monitoring biomarker for HNSCC, particularly in LSCC patients.

## 4. Discussion

As key mediators of intercellular communication, sEVs play a role in shaping the tumour microenvironment, reflected by tumour progression and metastatic formations [18,39]. In turn, cancer cells continuously secrete sEVs with specific cargo, including tumour-associated proteins, tetraspanins, and integrins, which can enhance motility and metastasis, allowing tumour cells to reprogram the surrounding microenvironment [40,41]. In this study, we identified vWF in plasma-derived sEVs as a potential biomarker, effectively distinguishing HNSCC patients, particularly LSCC and OPSCC subtypes, from benign individuals. However, the OPSCC was relatively uncommon in our study cohort; disease stage-based comparisons of the OPSCC were in a relatively small sample size [4]. Therefore, both its apparent diagnostic performance and the observed increase in the incidence of early-stage OPSCC should be considered exploratory and will require validation in a larger, independent cohort. Furthermore, longitudinal assessment of postoperative patients indicated that fluctuations in sEV-vWF abundance correlate with disease status and recurrence, underscoring its potential utility as a minimally invasive biomarker for not only diagnosis but also disease monitoring.

As an adhesive glycoprotein, under healthy conditions, vWF primarily plays a role in haemostasis by mediating platelet adhesion and stabilizing coagulation factor VIII. However, in cancer microenvironments, vWF has been shown to be involved in cancer-associated thrombosis and metastatic dissemination [42,43,44,45,46,47]. It has been reported that cancer cells induce normal endothelial cells to release vWF to establish a hypoxic or inflammatory environment [48,49].

Previous studies have detected vWF in cancer, cardiovascular disease, and depressive disorder plasma-derived sEVs, in which it is thought to be bound to the surface of sEVs. It has been reported that in these pathophysiological states, sEV-vWF exhibits distinct biological effects, different to that of soluble, non-EV vWF [50,51]. The sEVs containing vWF contribute to the endothelial network formation, extracellular matrix remodelling, immune modulation, and angiogenesis during tumour progression [52,53,54,55]. This supports our findings of specific vWF enrichment in HNSCC sEVs compared to benign sEVs, suggesting active sorting and packaging of this protein into vesicular compartments during tumour development. Elevated vWF levels in cancer sEVs may also stem from stress-induced EV biogenesis in malignant cells [53,56]. Vesicular enrichment of vWF may reflect a mechanism by which cancer cells modulate the vascular niche and promote a pro-thrombotic microenvironment favourable for metastasis [47,57,58].

The non-differentiating abundances of vWF in total plasma highlight the superior disease specificity of sEV-biomarker profiling compared to conventional plasma testing, as plasma may potentially obscure subtle cancer-associated alterations due to the abundance of circulating proteins from systemic inflammation or vascular pathology [59,60]. This validates the clinical value of investigating individual sEV proteomes and other genetic content (including RNAs and lipids) rather than relying solely on whole plasma analysis. Notably, similar findings have been observed in other malignancies, such as pancreatic, lung [61], and hepatocellular carcinoma [62], where the diagnostic accuracy of sEV proteins surpassed that of their soluble counterparts. However, the mechanisms by which cancer-derived sEVs specifically enrich for vWF, and whether vWF enrichment is specific to cancer sEVs, are still unknown. Other confounding factors such as age, inflammation, vascular activation, and hormonal status also impact vWF expression and abundance [63,64,65,66]. Although the age-stratified analysis supported an association between elevated sEV-vWF levels and HNSCC, the age matching between the benign group and the HNSCC group was not ideal, and the limited sample size of the elderly benign group (>60 years) restricted the ability to completely rule out age as a potential confounding factor. Hence, large-scale cohort studies with appropriate controls matched by clinical covariates are strictly required in the future.

HPV status can be used to classify HNSCC into different biological and clinical subtypes, particularly in the oropharynx [7,38]. As HPV data were not systematically collected in this study cohort, it was not possible to perform subgroup analyses. This should be addressed in future validation studies.

Expanding beyond cancer detection, sEV biomarkers may serve as indicators for early cancer recurrence. Longitudinal monitoring in our study suggests that dynamic changes in sEV-vWF abundance reflects treatment response and disease recurrence, consistent with previous findings in glioblastoma, hepatocellular carcinoma, and gastric cancer [51,53,67]. The decline in sEV-vWF abundance following surgical tumour resection, coupled with its subsequent elevation in recurrent patients, supports the notion that plasma sEV cargo can reflect disease activity in real time. Consequently, regular monitoring of plasma sEV-vWF may complement existing imaging and histopathological approaches, offering a less invasive and more responsive surveillance modality [68,69,70].

## 5. Conclusions

In summary, our study has identified vWF as a novel sEV biomarker for distinguishing HNSCC from benign head and neck disease, demonstrating particular significance in LSCC and OPSCC. Beyond diagnostic value, the correlation between pre- and post-operative sEV-vWF dynamics and recurrence rates indicates the potential utility of vWF in disease monitoring. These findings support the integration of sEV-based liquid biopsy approaches into future HNSCC clinical trials, potentially offering novel pathways for early detection, personalised monitoring, and enhanced patient management.

## Figures and Tables

**Table 1:** Clinicopathology Information.

	Total		Benign		OCSCC		HSCC		LSCC		OPSCC	
***N***	96		25		24		12		28		7	
**Sex, *n* (%)**												
Male	81	87.50%	22	88.00% ^ns^	16	66.67%	11	91.67%	27	96.43%	5	71.43%
Female	14	29.17%	3	12.00%	8	33.33%	^#^		1	3.57%	2	28.57%
**Age, y**												
Median (range)	59 (25, 86)		39 (25, 70) ***		63.5 (41, 86)		64 (50, 72)		67 (45, 81)		60.5 (44, 76)	
**Staging (AJCC)**	71											
I	12	16.90%	NA		7	29.17%	3	25.00%	NA		2	28.57%
II	12	16.90%			5	20.83%	2	16.67%	3	10.71%	2	28.57%
III	11	15.49%			3	12.50%	1	8.33%	7	25.00%	NA	
IVA	33	46.48%			8	33.33%	4	33.33%	18	64.29%	3	42.86%
IVB	3	4.23%			1	4.17%	2	16.67%	NA		NA	
**T stage**	71											
Tis	6	8.45%			1	4.17%	4	33.33%	NA		1	14.29%
T1	13	18.31%			11	45.83%	NA		NA		2	28.57%
T2	4	5.63%			2	8.33%	1	8.33%	NA		1	14.29%
T3	15	21.13%			2	8.33%	2	16.67%	11	39.29%	NA	
T4a	31	43.66%			7	29.17%	4	33.33%	17	60.71%	3	42.86%
T4b	2	2.82%			1	4.17%	1	8.33%	NA		NA	
**N stage**	71											
N0	41	57.75%			15	62.50%	5	41.67%	15	53.57%	6	85.71%
N1	7	9.86%			NA		2	16.67%	5	17.86%	NA	
N2a	2	2.82%			1	4.17%	NA		1	3.57%	NA	
N2b	10	14.08%			8	33.33%	NA		2	7.14%	NA	
N2c	7	9.86%			NA		2	16.67%	5	17.86%	NA	
N3a	1	1.41%			NA		1	8.33%	NA		NA	
N3b	3	4.23%			NA		2	16.67%	NA		1	14.29%
N3c	0	0.00%			NA		NA		NA		NA	
**HPV Status**												
p16+	4		NA		2		NA		1		1	
p16−	14				6		3		2		3	
**Smoking**	68											
Non-smoker	20	29.41%	NA		12	52.17%	1	10.00%	4	14.29%	3	42.86%
Current smoker	28	41.18%			3	13.04%	4	40.00%	18	64.29%	3	42.86%
Ex-smoker	20	29.41%			8	34.78%	5	50.00%	6	21.43%	1	14.29%
**Alcohol**	71											
Non-drinker	27	38.03%	NA		12	50.00%	3	25.00%	9	32.14%	3	42.86%
Current drinker	30	42.25%			7	29.17%	6	50.00%	14	50.00%	3	42.86%
Ex-drinker	14	19.72%			5	20.83%	3	25.00%	5	17.86%	1	14.29%

*** *p*-value < 0.001, ^ns^ not significant. ^#^ Gender information for one HSCC patient is missing. OCSCC: oral cavity squamous cell carcinoma; HSCC: hypopharyngeal squamous cell carcinoma; LSCC: laryngeal squamous cell carcinoma; OPSCC: oropharyngeal squamous cell carcinoma.

## Data Availability

All data generated or analysed during this study are included in this published article and Supplementary Materials.

## Supplementary Materials

The following supporting information can be downloaded at: https://www.mdpi.com/article/10.3390/cancers18142339/s1, Figure S1: Proteomic analysis of plasma-derived sEVs from benign individuals and HNSCC patients. Heatmap showing the relative expression levels of all quantified proteins across sEVs derived from benign individuals and HNSCC patients. The color key denotes the row z-score. HNSCC: head and neck squamous cell carcinoma; Figure S2: Proteins identified as differentially expressed by proteomic analysis showed no significant differences when validated by ELISA. Quantification of THBS1 (A), MAC-2BP (B), NID1 (C), SDC1 (D), LBP (E), and HMCN1 (F) in plasma-derived sEVs from individuals with benign head and neck disease and HNSCC patients, measured by ELISA. Data are shown as mean ± SEM, ns, not significant. Statistical analyses were performed using unpaired *t*-test. HNSCC: head and neck squamous cell carcinoma; Figure S3: Age-stratified analysis of plasma sEV-vWF levels in benign controls and HNSCC patients. (A) sEV-vWF levels in benign and HNSCC patients stratified by age. (B) Correlation analysis between age and sEV-vWF levels in benign-only, HNSCC patient-only, and HNSCC combined with benign. (C) sEV-vWF levels in benign and HNSCC subgroups stratified by age (<60 years and ≥60 years). Data are shown as mean ± SEM, * *p* < 0.05. Statistical analyses were performed using unpaired *t*-test, nonparametric Spearman correlation. HNSCC: head and neck squamous cell carcinoma; OCSCC: oral cavity squamous cell carcinoma; HSCC: hypopharyngeal squamous cell carcinoma; LSCC: laryngeal squamous cell carcinoma; OPSCC: oropharyngeal squamous cell carcinoma; Figure S4: ELISA-based validation showed no significant difference in sEV-vWF in HSCC and OCSCC patients compared to benign individuals, and no significant differences according to HPV (p16) status. Validation of sEV-vWF in individuals with (A) benign head and neck disease (*n* = 25) compared to HSCC patients (*n* = 12) and (B) benign head and neck disease (*n* = 25) compared to OCSCC patients (*n* = 24). (C) Validation of sEV-vWF in HNSCC patients tested as p16-negative for HPV (*n* = 14) compared to p16-positive patients (*n* = 4). Data are shown as mean ± SEM, ns, not significant. Statistical analyses were performed using unpaired *t*-test. HSCC: hypopharyngeal squamous cell carcinoma; OCSCC: oral cavity squamous cell carcinoma; HNSCC: head and neck squamous cell carcinoma; Table S1: Full List of Proteins Identified by Mass Spectrometry; File S1: Original WB figures and Greyscale analysis.

## Abbreviations

The following abbreviations are used in this manuscript:

AUC	Area Under the Curve
CT	Computed Tomography
DSG1	Desmoglein-1
HMCN1	Hemicentin-1
HNC	Head and Neck Cancer
HNSCC	Head and Neck Squamous Cell Carcinoma
HPV	Human Papillomavirus
HSCC	Hypopharyngeal Squamous Cell Carcinoma
IRE1	Inositol Requiring Enzyme 1
LBP	Lipopolysaccharide Binding Protein
LSCC	Laryngeal Squamous Cell Carcinoma
MAC-2BP	Mac-2 Binding Protein
MRI	Magnetic Resonance Imaging
NID1	Nidogen-1
NTA	Nanoparticle Tracking Analysis
OCSCC	Oral Cavity Squamous Cell Carcinoma
OPSCC	Oropharyngeal Squamous Cell Carcinoma
SEVs	Small extracellular vesicles
SDC1	Syndecan-1
THBS1	Thrombospondin-1
vWF	Von Willebrand factor
