# Supplementary material for: Identification of von Willebrand Factor-Enriched Small Extracellular Vesicles as a Blood-Based Biomarker for the Detection of Head and Neck Squamous Cell Carcinoma
Source: Cancers (Basel). 2026 Jul 20;18(14):2339. doi: 10.3390/cancers18142339 (PMC13406140; doi:10.3390/cancers18142339)
Supplement: Supplementary file 1 [file cancers-18-02339-s001.zip › File S1. Original WB figures and Greyscale analysis.pptx]

## Slide 1
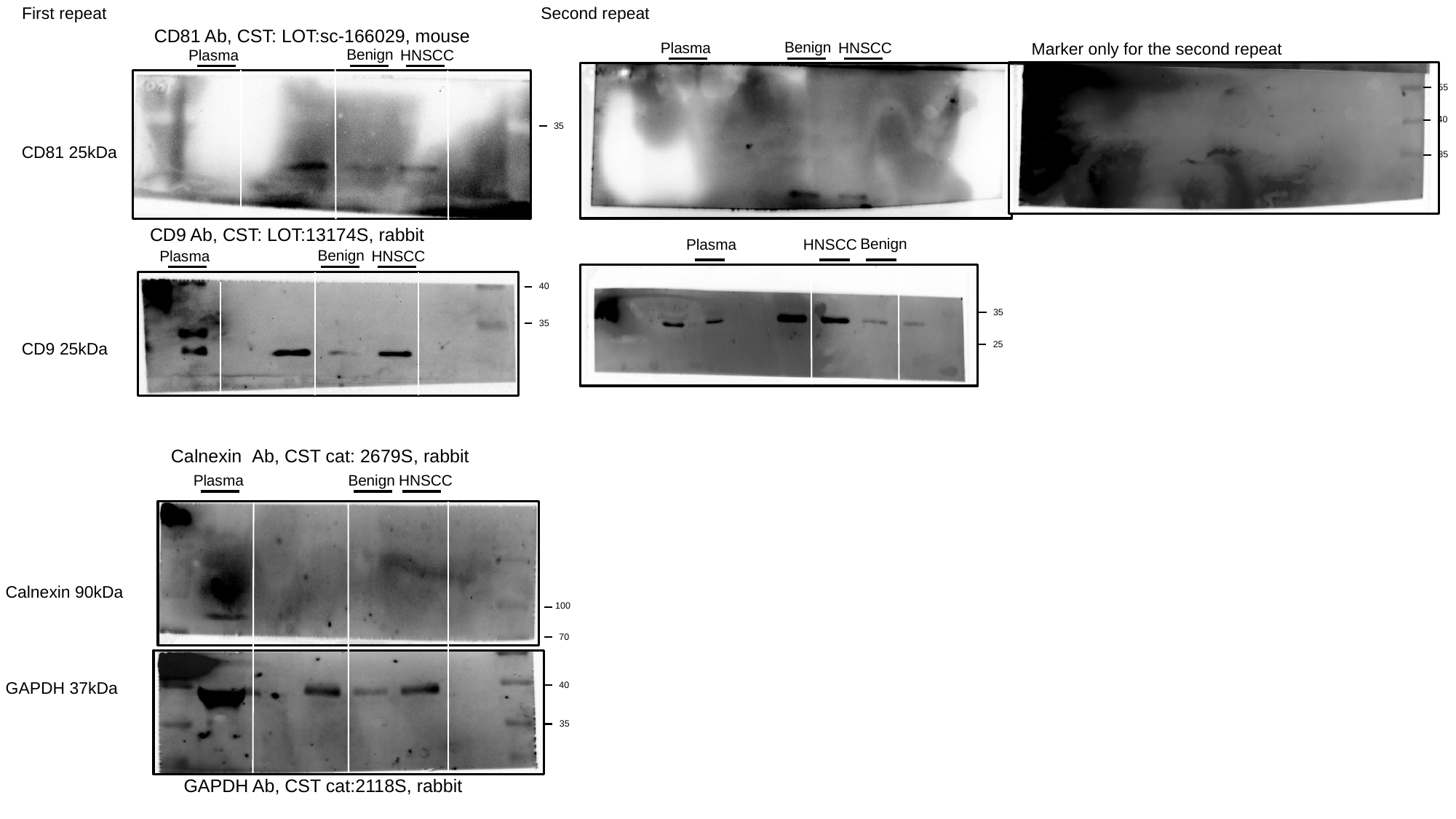

First repeat
Second repeat
CD81 Ab, CST: LOT:sc-166029, mouse
Benign
Plasma
HNSCC
Marker only for the second repeat
Benign
Plasma
HNSCC
55
40
35
CD81 25kDa
35
CD9 Ab, CST: LOT:13174S, rabbit
Benign
Plasma
HNSCC
Benign
Plasma
HNSCC
40
35
35
CD9 25kDa
25
Calnexin Ab, CST cat: 2679S, rabbit
Benign
Plasma
HNSCC
Calnexin 90kDa
100
70
GAPDH 37kDa
40
35
GAPDH Ab, CST cat:2118S, rabbit

## Slide 2
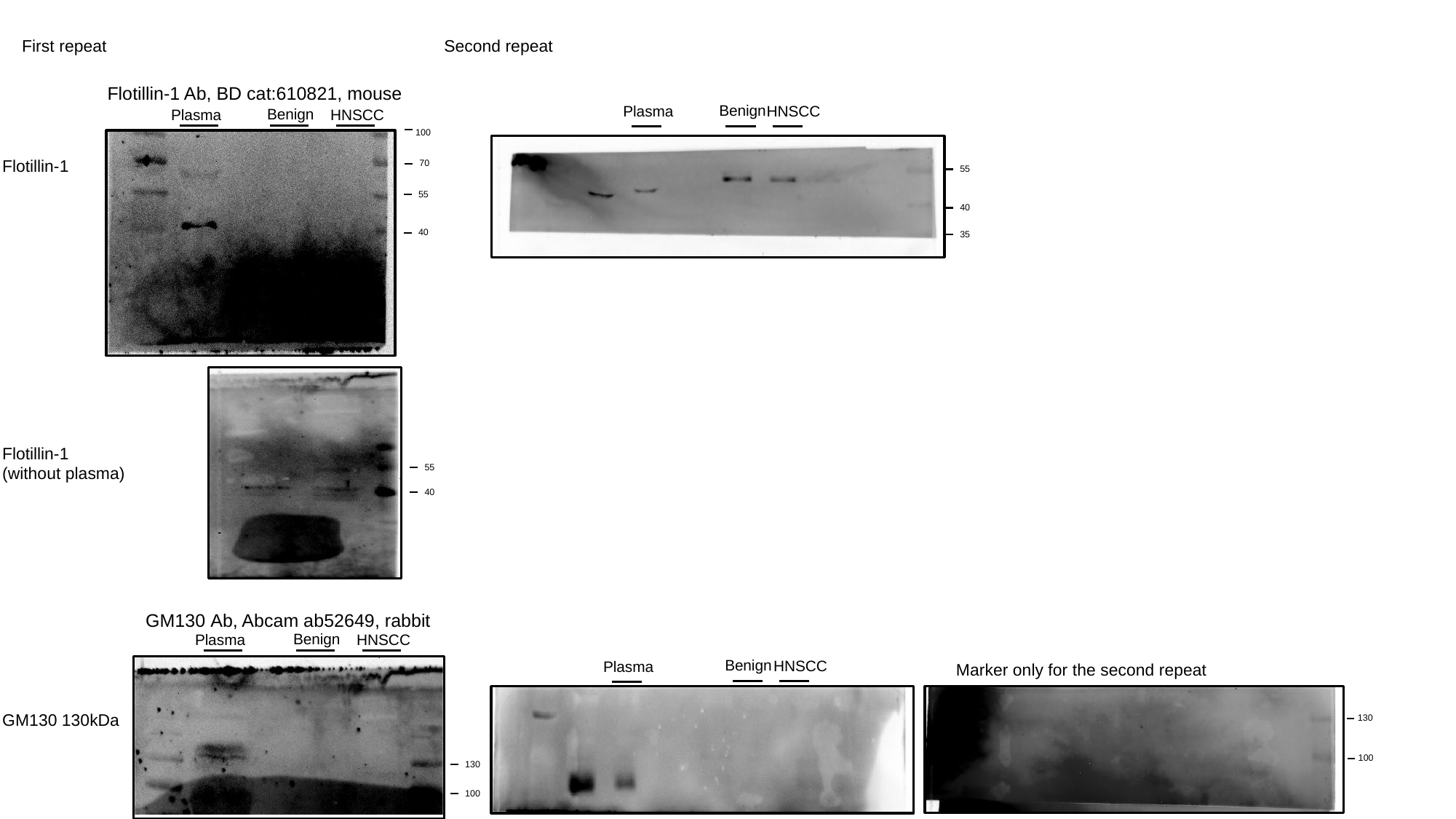

First repeat
Second repeat
Flotillin-1 Ab, BD cat:610821, mouse
Benign
Plasma
HNSCC
Benign
Plasma
HNSCC
100
Flotillin-1
70
55
55
40
40
35
Flotillin-1 (without plasma)
55
40
GM130 Ab, Abcam ab52649, rabbit
Benign
Plasma
HNSCC
Benign
HNSCC
Plasma
Marker only for the second repeat
GM130 130kDa
130
100
130
100

## Slide 3
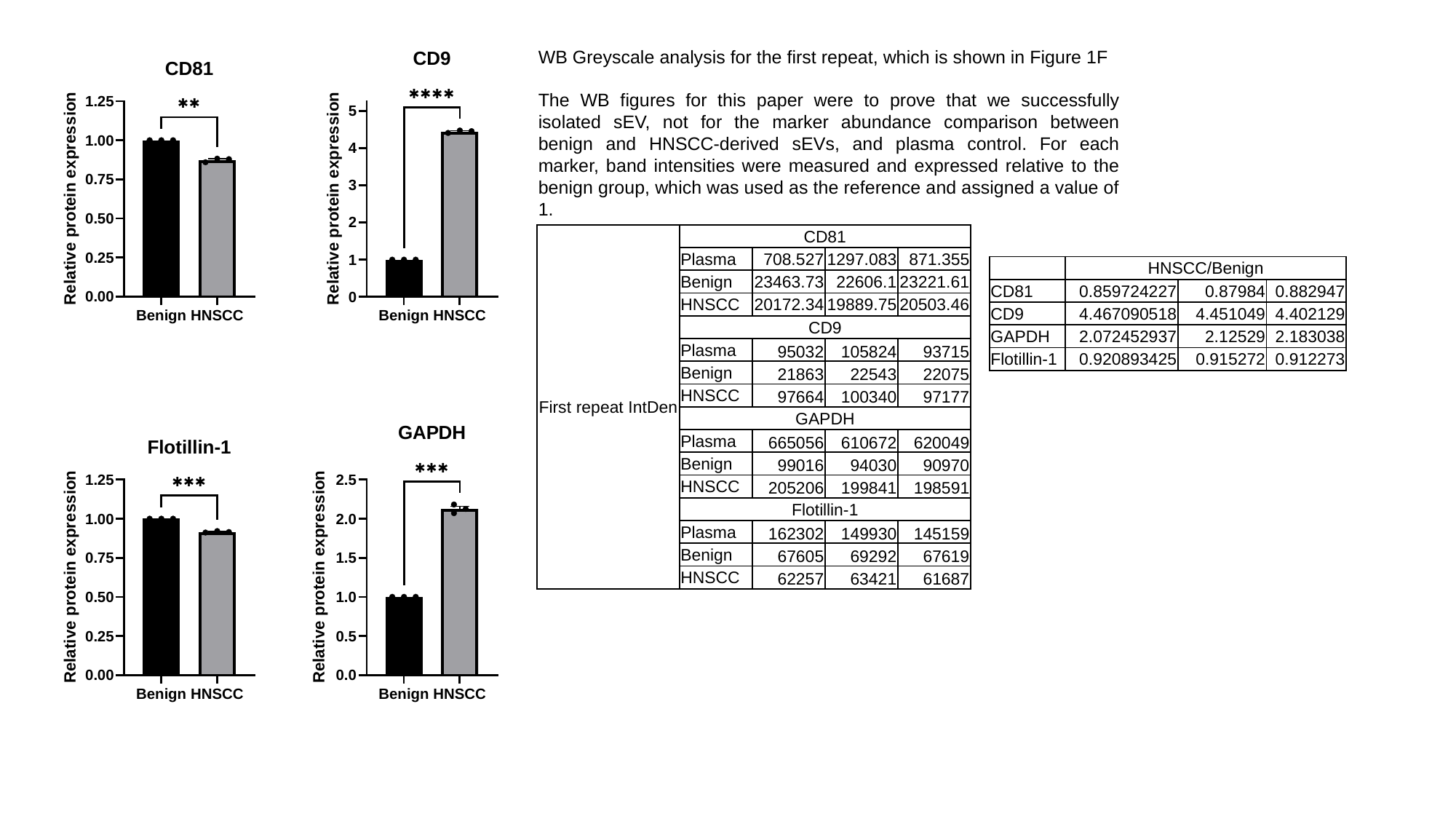

WB Greyscale analysis for the first repeat, which is shown in Figure 1F
The WB figures for this paper were to prove that we successfully isolated sEV, not for the marker abundance comparison between benign and HNSCC-derived sEVs, and plasma control. For each marker, band intensities were measured and expressed relative to the benign group, which was used as the reference and assigned a value of 1.
| First repeat IntDen | CD81 | | | |
| --- | --- | --- | --- | --- |
| | Plasma | 708.527 | 1297.083 | 871.355 |
| | Benign | 23463.73 | 22606.1 | 23221.61 |
| | HNSCC | 20172.34 | 19889.75 | 20503.46 |
| | CD9 | | | |
| | Plasma | 95032 | 105824 | 93715 |
| | Benign | 21863 | 22543 | 22075 |
| | HNSCC | 97664 | 100340 | 97177 |
| | GAPDH | | | |
| | Plasma | 665056 | 610672 | 620049 |
| | Benign | 99016 | 94030 | 90970 |
| | HNSCC | 205206 | 199841 | 198591 |
| | Flotillin-1 | | | |
| | Plasma | 162302 | 149930 | 145159 |
| | Benign | 67605 | 69292 | 67619 |
| | HNSCC | 62257 | 63421 | 61687 |
| | HNSCC/Benign | | |
| --- | --- | --- | --- |
| CD81 | 0.859724227 | 0.87984 | 0.882947 |
| CD9 | 4.467090518 | 4.451049 | 4.402129 |
| GAPDH | 2.072452937 | 2.12529 | 2.183038 |
| Flotillin-1 | 0.920893425 | 0.915272 | 0.912273 |
