# Supplementary material for: Identification of von Willebrand Factor-Enriched Small Extracellular Vesicles as a Blood-Based Biomarker for the Detection of Head and Neck Squamous Cell Carcinoma
Source: Cancers (Basel). 2026 Jul 20;18(14):2339. doi: 10.3390/cancers18142339 (PMC13406140; doi:10.3390/cancers18142339)
Supplement: Supplementary file 1 [file cancers-18-02339-s001.zip › Supplementary Figures.pdf]

Identification of von Willebrand Factor-Enriched Small Extracellular Vesicles as a Blood-Based Biomarker for the Detection of Head and Neck Squamous Cell Carcinoma

Yue Su, Kekoolani S. Visan, Sunyoung Ham, Xuanxuan Li, Su-Ho Park, Cherrie W. K. Ng, Judy Wai Ping Yam, Jason Y. K. Chan and Andreas Möller

Supporting figures

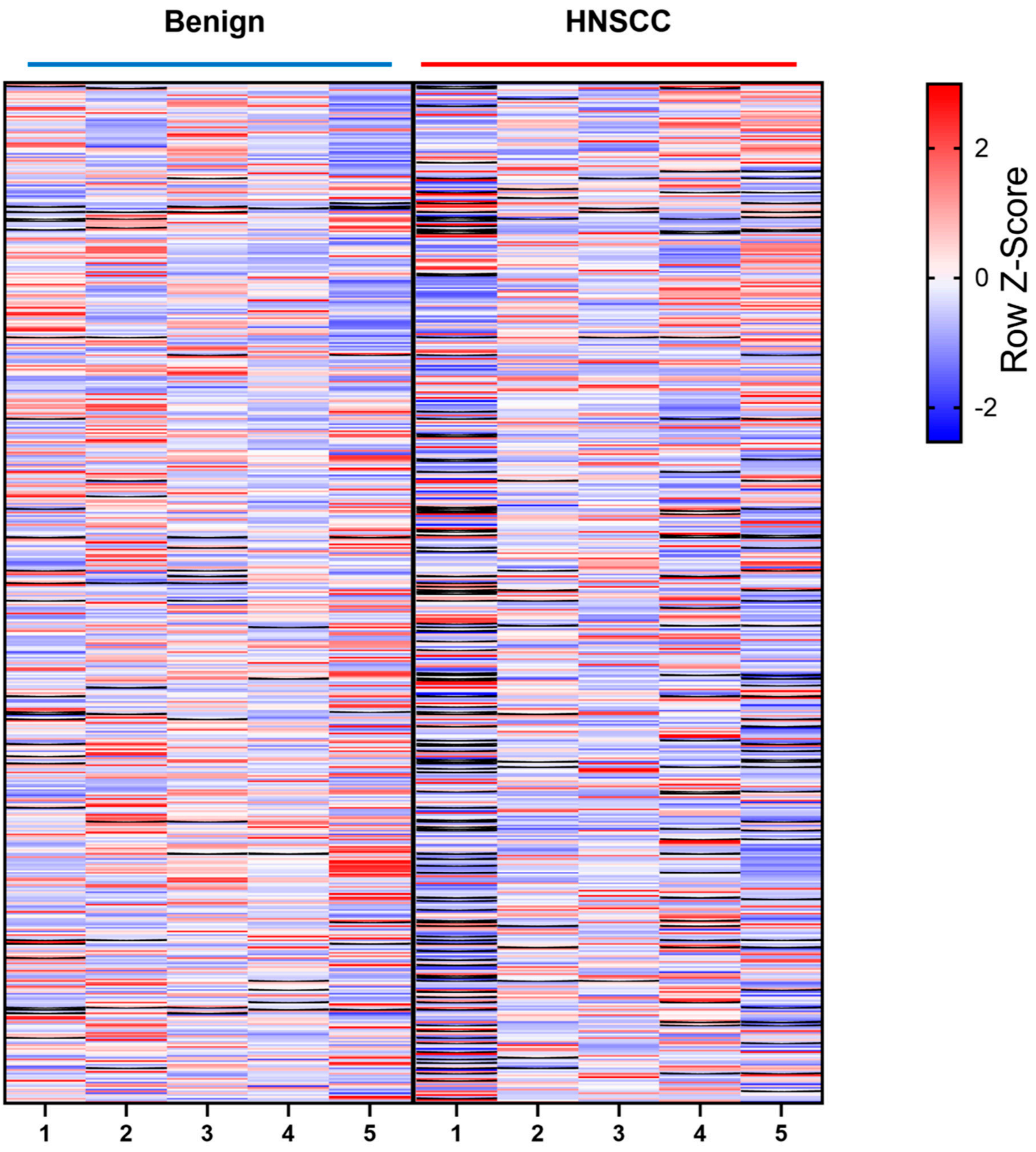

Figure S1. Proteomic analysis of plasma-derived sEVs from benign individuals and HNSCC patients. Heatmap showing the relative expression levels of all quantified proteins across sEVs derived from benign individuals and HNSCC patients. The color key denotes the row z-score. HNSCC: head and neck squamous cell carcinoma.

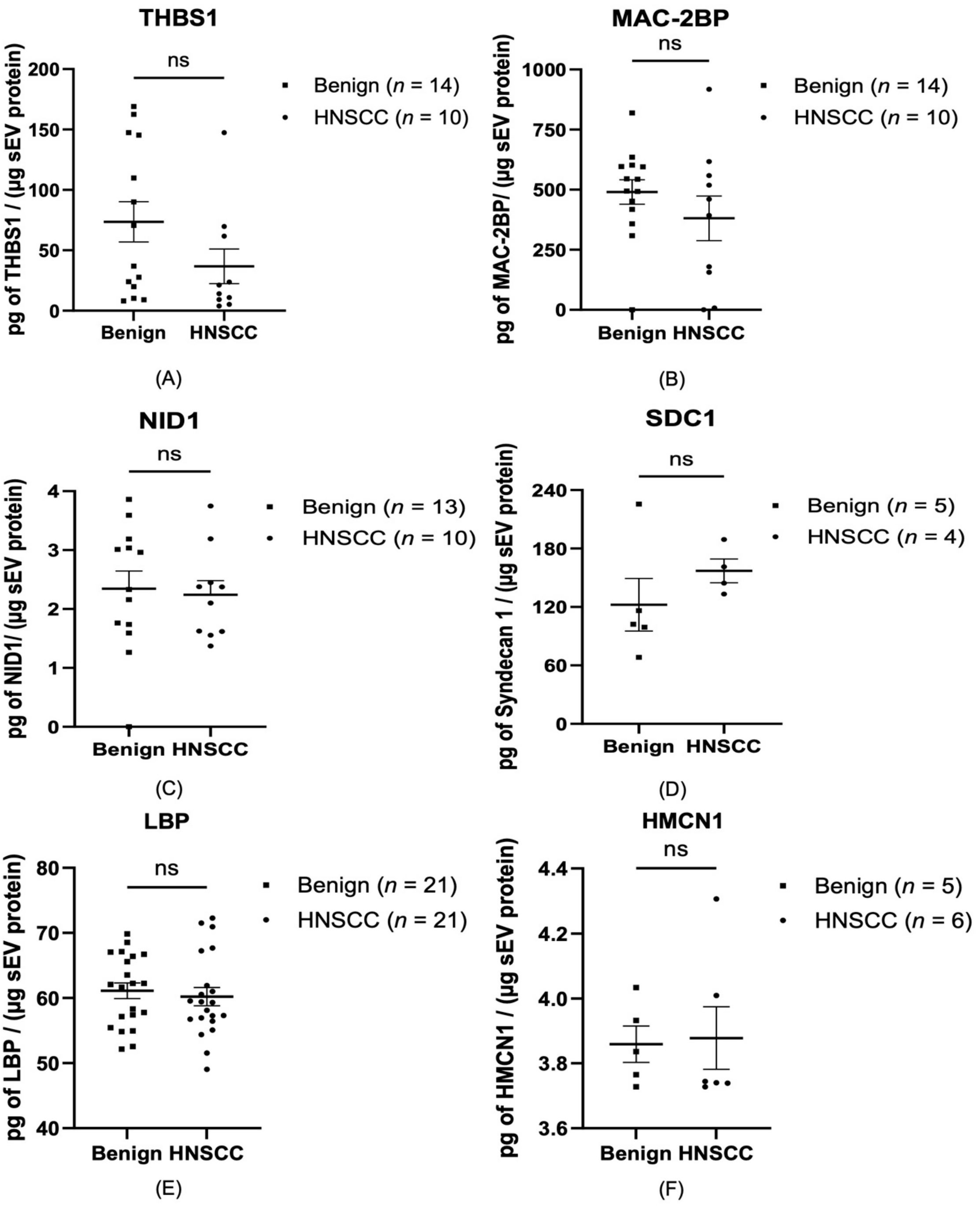

Figure S2. Proteins identified as differentially expressed by proteomic analysis showed no significant differences when validated by ELISA. Quantification of THBS1 (**A**), MAC-2BP (**B**), NID1 (**C**), SDC1 (**D**), LBP (**E**), and HMCN1 (**F**) in plasma-derived sEVs from individuals with benign head and neck disease and HNSCC patients, measured by ELISA. Data are shown as mean ± SEM, ns, not significant. Statistical analyses were performed using unpaired *t*-test. HNSCC: head and neck squamous cell carcinoma.

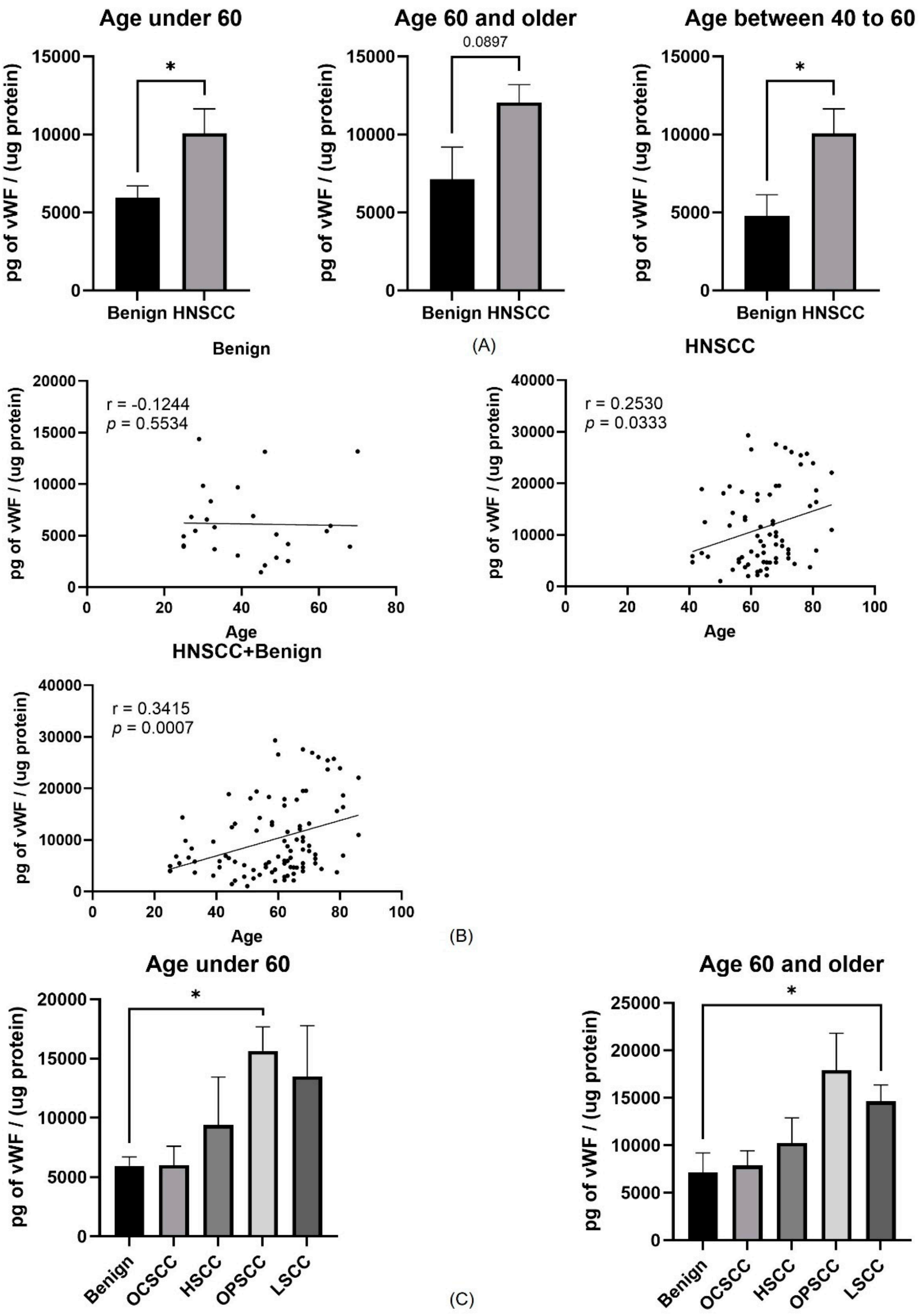

Figure S3. Age-stratified analysis of plasma sEV-vWF levels in benign controls and HNSCC patients. (**A**) sEV-vWF levels in benign and HNSCC patients stratified by age. (**B**) Correlation analysis between age and sEV-vWF levels in benign-only, HNSCC patient-only, and HNSCC combined with benign. (**C**) sEV-vWF levels in benign and HNSCC subgroups stratified by age (<60 years and ≥60 years). Data are shown as mean ± SEM, * $p < 0.05$. Statistical analyses were performed using unpaired *t*-test, nonparametric Spearman correlation. HNSCC: head and neck squamous cell carcinoma; OCSCC: oral cavity squamous cell carcinoma; HSCC: hypopharyngeal squamous cell carcinoma; LSCC: laryngeal squamous cell carcinoma; OPSCC: oropharyngeal squamous cell carcinoma.

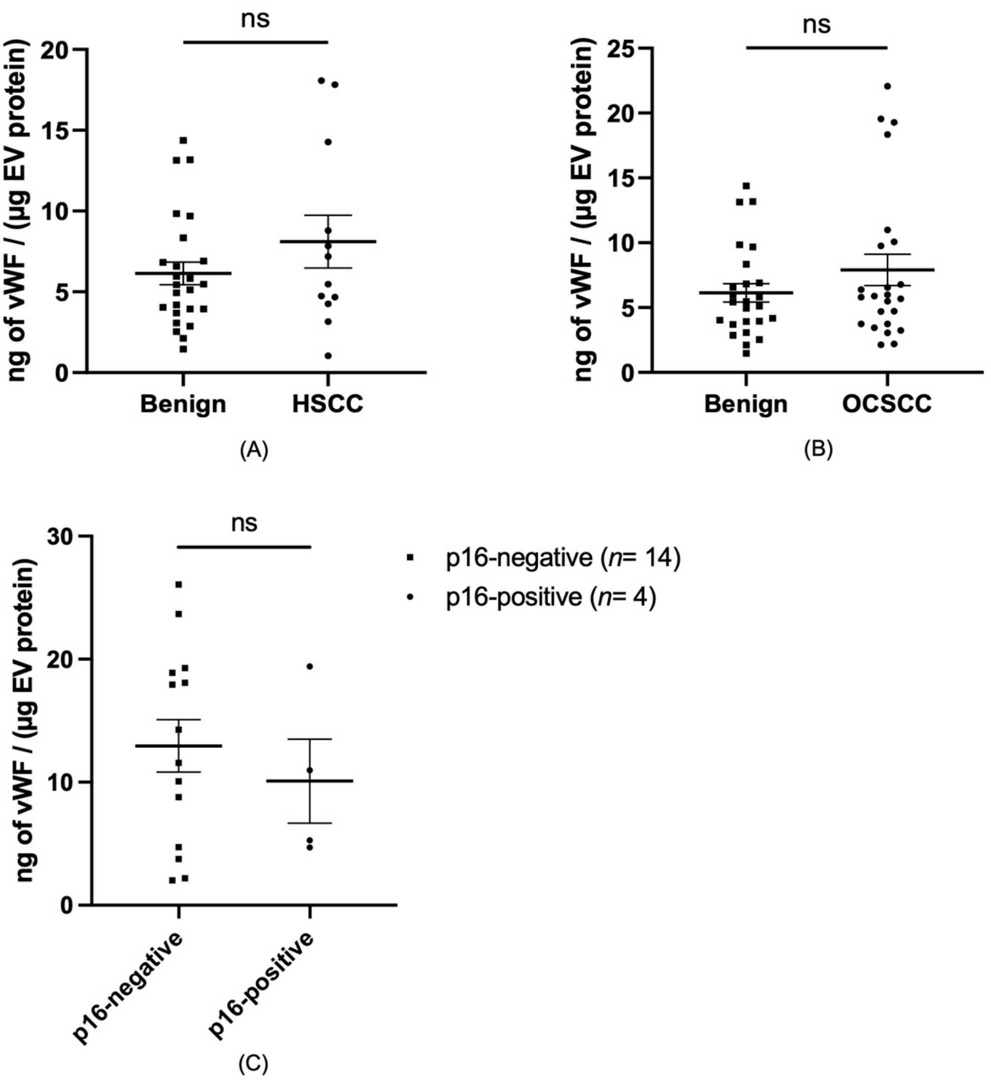

Figure S4. ELISA-based validation showed no significant difference in sEV-vWF in HSCC and OCSCC patients compared to benign individuals, and no significant differences according to HPV (p16) status. Validation of sEV-vWF in individuals with (**A**) benign head and neck disease (n = 25) compared to HSCC patients (n = 12) and (**B**) benign head and neck disease (n = 25) compared to OCSCC patients (n = 24). (**C**) Validation of sEV-vWF in HNSCC patients tested as p16-negative for HPV (n = 14) compared to p16-positive patients (n = 4). Data are shown as mean ± SEM, ns, not significant. Statistical analyses were performed using unpaired t-test. HSCC: hypopharyngeal squamous cell carcinoma; OCSCC: oral cavity squamous cell carcinoma; HNSCC: head and neck squamous cell carcinoma.
